# The efficacy of paxlovid in elderly patients infected with SARS-CoV-2 omicron variants: Results of a non-randomized clinical trial

**DOI:** 10.3389/fmed.2022.980002

**Published:** 2022-09-06

**Authors:** Weijie Zhong, Xiufeng Jiang, Xiaosheng Yang, Tiantong Feng, Zhixin Duan, Wei Wang, Zhaoliang Sun, Lingyan Chen, Xin Nie, Chuanlong Zhu, Wenchuan Zhang, Yi Li

**Affiliations:** ^1^Department of Neurosurgery, Ninth People’s Hospital Affiliated to Shanghai Jiao Tong University School of Medicine, Shanghai, China; ^2^Department of Infectious Disease, The First Affiliated Hospital of Nanjing Medical University, Nanjing, China; ^3^Clinical Research Unit, Shanghai Ninth People’s Hospital, Shanghai Jiao Tong University School of Medicine, Shanghai, China; ^4^Biostatistics Office of Clinical Research Unit, Shanghai Ninth People’s Hospital, Shanghai Jiao Tong University School of Medicine, Shanghai, China

**Keywords:** COVID-19, paxlovid, nucleic acid shedding time, omicron, SARS-CoV-2

## Abstract

**Objective:**

To evaluate the efficacy of Paxlovid in treating Chinese elder patients infected with SARS-CoV-2 omicron variants.

**Materials and methods:**

We performed a non-randomized, controlled trial in Shanghai, China. Participants infected with SARS-CoV-2 omicron variants were enrolled. All patients were divided into the Paxlovid group or the control group according to the Chinese guideline (version 9). The nucleic acid shedding time was the primary endpoint.

**Results:**

According to the inclusion criteria, 142 patients infected with omicron variants were enrolled, 36 patients who did not receive paxlovid were assigned to the control group, and 106 were in the Paxlovid group. The baseline characteristics were similar in either group. No significant difference in BMI, age, time from onset to patient enrollment, the severity on first admission, vaccination status, comorbidity, first symptoms, and laboratory results were recorded. Compared to the control group, participants in the Paxlovid group had a shorter viral shedding time [11.11 (2.67) vs. 9.32 (2.78), *P* = 0.001].

**Conclusion:**

In Chinese elder patients infected with the variant of SARS-CoV-2 omicron, our data suggest that Paxlovid can significantly reduce the nucleic acid shedding time.

## Graphical Abstract

Clinical trial flow chart. 
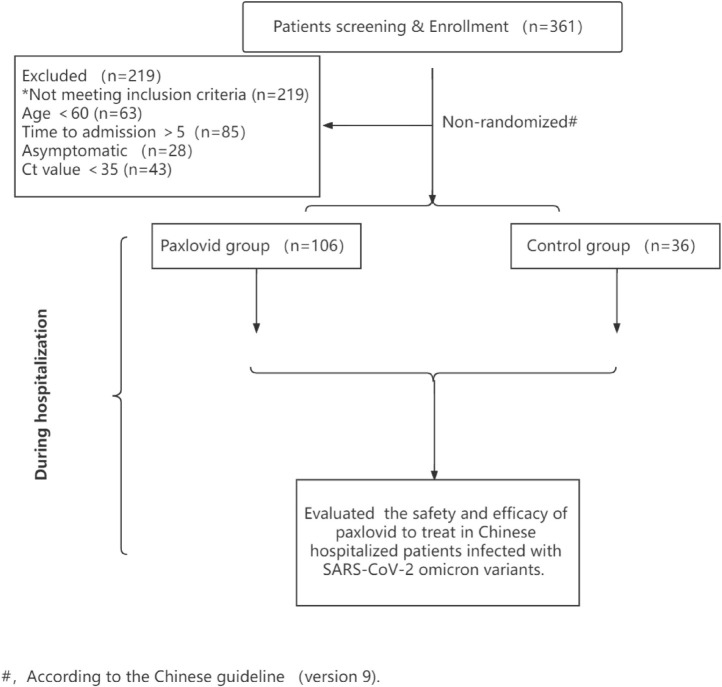

## Introduction

The coronavirus disease 2019 (COVID-19) pandemic is one of the greatest threats to human health in the 21st century (1–3). With its high contagious capacity, over 500 million cases were confirmed worldwide (WHO). During the three years of the battle between humans and viruses, the SARS-CoV-2 variants have also been constantly updated (4–6). Currently, the SARS-CoV-2 omicron variant has become the predominant variant circulating in the world (4, 7–9). After comparing the genomes of viruses that broke out in Shanghai, China, in 2022, it was found that the genomes of the newly infected viruses in Shanghai belong to the BA.2.2 sub-lineages. It is worth noting that BA.2 is a sub-strain of the omicron variant (B.1.1.159) (10, 11).

Different from the characteristics of the previous variants of SARS-CoV-2, evidence confirms that the omicron variant is less severe than previous variants, and the severity or mortality rate of elderly patients is higher than that of the general population (12, 13). The reported case fatality rate for people over 60 years old (about 19.30% of people in this age group are not vaccinated) is 2.70%. In May 2022, a report titled “New versions of Omicron are masters of immune evasion” on the front page of the Science journal considered that based on the immunological characteristics of the omicron variant, it is recommended to define it as SARS-CoV-3, a virus different from SARS-CoV-2. This conclusion has not been unified, but it is worth noting that omicron variants lead to widespread escape of existing neutralizing antibodies and increased vaccine breakthrough rates based on hyper-mutation of the spike protein (14–16). The surprising immune evasion ability of the omicron variant may bring many challenges to a specific drug or vaccine development (17, 18).

Therefore, the effectiveness of specific drugs developed in the past may vary due to different virus variants. Nirmatrelvir/ritonavir (Paxlovid) has received the emergency use authorization (EUA) for the treatment of patients with SARS-CoV-2 (19). It has been approved for use in many regions. According to reports, its intervention effect in the COVID-19 is as high as 87% (20). *In vitro* studies found that Paxlovid retains activity against the omicron variant (21). Nevertheless, clinical studies on the efficacy of Paxlovid in Chinese patients infected with the SARS-CoV-2 omicron are still lacking.

Among the known variants, the omicron variant is the most infectious (4). Older people are a high-risk factor for exacerbating of the disease after infecting with omicron. Additionally, with the increasing number of deaths and cases worldwide, it is significant to intensify the study of COVID-19 infected by the omicron variant. Based on this, we conducted a non-randomized trial to assess the safety and efficacy of Paxlovid to treat in Chinese elder patients infected with omicron variants.

## Materials and methods

### Patients and oversight

From April 24 to May 28, 2022, a total of 142 patients with SARS-CoV-2 omicron variants were enrolled according to the inclusion criteria. All patients were referred from the Ninth People’s hospital, Shanghai Jiao Tong University School of Medicine. The inclusion criteria were: (1) Either male or female (60 years or older), diagnosed with SARS-CoV-2 infection without receiving systematic treatment; (2) In line with the treatment principles of Paxlovid, including patients within five days of onset and patients of mild or moderate cases with high-risk factors for progression to severe cases; (3) Patients who agreed to use Paxlovid and did not have drug-drug interactions were enrolled in the Paxlovid group. (4) Patients who refused to use Paxlovid or had adverse drug reactions with Paxlovid recently were enrolled in the control group, such as amiodarone, carbamazepine, diazepam, and phenobarbital; (5) Voluntary informed consent. Exclusion criteria included: (1) Prior to current disease episode, any confirmed SARS-CoV-2 infection.

The study was approved by the ethics committee of the Ninth People’s Hospital, Shanghai Jiao Tong University School of Medicine (No. SH9H-2022-T112-2). Moreover, it was registered at the Chinese Clinical Trial Registry (ChiCTR2200060700).

### Trial design and procedures

This is a non-randomized trial to evaluate the safety and efficacy of Paxlovid in Chinese elder participants (60 years or older) infected with the variant of SARS-CoV-2 omicron. After subject consent from the participants, age, sex, time from onset to enrollment in patients, Ct values, disease history, disease severity at the first admission, initial-episode syndromes, comorbidities, vital signs, and vaccination status were collected for each patient at the baseline characteristics. After introducing the Paxlovid, patients were assessed for eligibility on the basis of the inclusion and exclusion criteria (Graphical Abstract).

A total of 106 eligible candidates were assigned to the Paxlovid group that received 300mg nirmatrelvir and 100mg ritonavir for 12 h for 5 days. Others were assigned to the control group that received standard of care for COVID-19. The discharge criteria were as follow: (1) normal body temperature for at least three consecutive days; (2) Respiratory symptoms and pulmonary imaging improved significantly; (3) Nucleic acid tests were negative twice consecutively for at least 24 h. Before their discharge, clinical study information collected for each patient included nucleic acid shedding time, time from symptom appearance to the disappearance, severe cases rate during hospitalization, laboratory results, adverse events, and mortality. All the patients were monitored by clinicians daily in our unit before their discharge and received a standard treatment regimen based on the Chinese guideline (version 9).

### Outcome measures

We considered time to viral clearance as the primary endpoint. After enrollment, COVID-19 was diagnosed by reverse transcription-polymerase chain reaction (RT-PCR) using serial nasopharyngeal swab specimens and once every day since administration. The criteria of nucleic acid shedding are according to Chinese guidelines (version 9), including (1) the N and ORF1ab gene are less than 35; (2) Two consecutive negative tests; (3) Interval between two consecutive tests is at least 24 h apart.

Secondary endpoints were time from symptom appearance to the disappearance, laboratory changes, severe cases rate during hospitalization, and mortality.

Safety endpoint was to assess the adverse events during the hospital admission. It refers to unforeseen medical events that occur when the patients receive administration. The researcher regularly assessed the patient’s symptoms and vital signs and documented adverse events.

### Statistical analysis

Continuous variables were presented as mean (Standard Deviation, SD) or median (Min-Max) and categorical variables were presented as numbers (%). Continuous variables were compared with Mann-Whitney U test or *t*-tests, and categorical variables were compared by χ^2^ test or Fisher’s exact tests. After that, The nucleic acid shedding time was developed using the Kaplan-Meier method. Statistical significance for the study was defined as *P* ≤ 0.05.

## Results

During our study, 142 hospitalized elder patients infected with SARS-CoV-2 omicron variants were enrolled, including 36 in the control group and 106 in the Paxlovid group (Graphical Abstract).

The characteristics of participants are summarized in Table 1. Baseline characteristics of all participants, including BMI, age, sex, time from onset to enrollment in patients, the severity on the first admission, vaccine, comorbidity, first symptoms, and laboratory results, were recorded **(Table 1**).

**TABLE 1:** Baseline characteristic of participations at the first admission.

Characteristics	Total (*N* = 142)	Control group (*N* = 36)	Paxlovid group (*N* = 106)	*P*-value	*P*-value*
Age, mean (SD), year	76.37 (9.70)	76.58 (9.77)	76.30 (9.72)	0.881	0.888
BMI, mean (SD), kg/m^2^	23 (2.15)	23.02 (3.08)	22.99 (3.19)	0.965	0.940
CT.N, mean (SD)^a^	28.69 (2.91)	28.88 (2.78)	28.62 (2.97)	0.656	0.826
CT.ORF, mean (SD)	27.97 (3.21)	28.29 (2.97)	27.86 (3.36)	0.501	0.442
**Sex**				0.611	–
Male, n (%)	58 (40.84%)	16 (44.44%)	42 (39.62%)		
Female, n (%)	84 (59.15%)	20 (55.56%)	64 (60.38%)		
Time from onset to enrollment in patients, median (Min-Max), day^b^	1 (0–5)	1 (0–5)	1 (0–5)	0.147	–
**Degree^c^**				0.440	–
Mild cases, n (%)	118 (83.10%)	32 (88.89%)	86 (81.13%)		
Moderate cases, n (%)	24 (16.90%)	4 (11.11%)	20 (18.87%)		
**Vaccine**				0.206	–
Unvaccinated, n (%)	95 (66.90%)	21 (58.33%)	74 (69.81%)		
Vaccinated, n (%)	47 (33.10%)	15 (41.67%)	32 (30.19%)		
**Comorbidity**					
Hypertension, n (%)	86 (60.56%)	23 (63.89%)	63 (59.43%)	0.637	–
Diabetes, n (%)	32 (22.54%)	11 (30.56%)	21 (19.81%)	0.247	–
Coronary artery disease, n (%)	56 (39.44%)	13 (36.11%)	43 (40.57%)	0.637	–
Stroke, n (%)	36 (25.35%)	10 (27.78%)	26 (24.53%)	0.699	–
Parkinson, n (%)	13 (9.15%)	2 (5.56%)	11 (10.38%)	0.516	–
Chronic pulmonary disease, n (%)	27 (19.01%)	4 (11.11%)	23 (21.70%)	0.162	–
Charlson, median(Min-Max)^d^	1 (0–7)	1 (0–3)	1 (0–7)	0.678	–
**First symptoms**					
Fever n (%)	69 (48.59%)	16 (45.71%)	53 (50.00%)	0.660	–
Fatigue, n (%)	34 (23.94%)	7 (19.44%)	27 (25.47%)	0.464	–
Cough, n (%)	120 (84.51%)	31 (86.11%)	89 (83.96%)	0.758	–
Expectoration, n (%)	95 (66.90%)	21 (58.33%)	74 (69.81%)	0.206	–
Sore throat, n (%)	57 (40.14%)	12 (33.33%)	45 (42.45%)	0.335	–
Nausea, n (%)	21 (14.79%)	4 (11.11%)	17 (16.04%)	0.472	–
Diarrhea, n (%)	19 (13.38%)	4 (11.11%)	15 (14.15%)	0.643	–
Abdominal pain, n (%)	10 (7.04%)	1 (2.78%)	9 (8.49%)	0.247	–
Headache, n (%)	13 (9.15%)	4 (11.11%)	9 (8.49%)	0.638	–
**Drug**					
Anticoagulation, n (%)	26 (18.31%)	6 (16.67%)	20 (18.87%)	0.768	–
Hormone, n (%)	19 (13.38%)	6 (16.67%)	13 (12.26%)	0.503	–
Chinese medicine, n (%)	137 (96.48%)	34 (94.44%)	103 (72.54%)	0.443	–
Antibiotic, n (%)	48 (33.80%)	12 (33.33%)	36 (33.96%)	0.945	–

*Indicated U test. ^a^Real-time PCR Ct value. ^b^Time from onset to enrollment in patients, including the time of initial symptoms or the first positive nucleic acid. ^c^According to WHO criteria. ^d^Charlson comorbidity index.

The average age of the 142 participants was 76.37 years, of which 118 (83.10%) were mild cases, and 24 (16.90%) were moderate cases. Among the 142 patients, 95 (66.90%) were vaccinated, and 47 (33.10%) were unvaccinated. The number of patients with hypertension was the largest, reaching 86 (60.56%). Regarding the first symptoms, more expectoration 95 (66.90%) and cough 120 (84.51%) were found than other symptoms.

The results of laboratory tests **(Table 2**) showed that there were 42 (29.58%) patients with decreased leukocyte (WBC<4 × 109/L) and 51 (35.92%) patients with decreased lymphocyte (L<1 × 109/L). In addition, there were also changes in hemoglobin and platelets. Among the biochemical indicators, the patients with decreased albumin (Albumin<35g/L) accounted for 14 (9.86%). No significant differences were found in the control group and the Paxlovid group in laboratory results.

**TABLE 2:** Laboratory results of patients with COVID-19 at enrollment and after treatment.

Characteristics	Total (*N* = 142)	Control group (*N* = 36)	Paxlovid group (*N* = 106)	*P*-value	*P*-value*
WBC mean (sd), 10^9^/L	4.90 (1.52)	5.10 (1.64)	4.82 (1.47)	0.350	0.594
L mean (sd), 10^9^/L	1.31 (0.59)	1.25 (0.47)	1.33 (0.63)	0.452	0.751
Hemoglobin mean (sd), g/L	129.21 (16.27)	127.61 (15.87)	129.75 (16.45)	0.497	0.479
Platelet count mean (sd), 10^9^/L	166.04 (48.03)	173.19 (44.41)	163.61 (49.16)	0.303	0.235
AST, median (Min-Max), U/L	17 (14–139)	25.50 (17–139)	26 (14–129)	0.105	0.654
ALT, median (Min-Max), U/L	26 (5–222)	17.50 (5–222)	16 (6–85)	0.873	0.460
Albumin mean (sd), g/L	39.38 (4.06)	39.72 (3.42)	39.26 (4.26)	0.560	0.693
CRP, median (Min-Max), mg/L	5.27 (0.08–267.01)	6.55 (1.27–66.80)	4.95 (0.08–267.01)	0.733	0.076
WBC<4.0, n (%)	42 (29.58%)	9 (25.00%)	33 (31.43%)	0.467	–
L<1.0, n (%)	51 (35.92%)	14 (38.89%)	37 (34.91%)	0.667	–
Platelet count <125 × 109/L, n (%)	22 (15.49%)	2 (5.56%)	20 (18.87%)	0.056	–
Hemoglobin <130 g/L, n (%)	78 (54.93%)	21 (58.33%)	57 (53.77%)	0.635	–
Albumin <35 g/L, n (%)	14 (9.86%)	1 (2.78%)	13 (12.26%)	0.009	–
CRP>10 mg/L, n (%)	44 (30.99%)	14 (40.00%)	30 (29.13%)	0.233	–

P* indicated U test. ALT, Alanine aminotransferase; AST, Aspartate aminotransferase; CRP, C-reactive protein; L, Lymphocyte count; WBC, White blood cell count.

Additionally, there were also no significant differences in BMI [23.02 (3.08) vs. 22.99 (3.19), *P* = 0.965], age [76.58 (9.77) vs. 76.30 (9.72), *P* = 0.881] and gender [16:20 vs. 42:64 (M: F), *P* = 0.611]. Moreover, there were no significant different in first symptoms and comorbidity, including fever [16 (45.71%) vs. 53 (50.00%), *P* = 0.660], fatigue [7 (19.44%) vs. 27 (25.47%), *P* = 0.464], cough [31 (86.11%) vs. 89 (83.96%), *P* = 0.758], expectoration, sore throat, hypertension [23 (63.89%) vs. 63 (59.43%), *P* = 0.637], diabetes [11 (30.56%) vs. 21 (19.81%), *P* = 0.247], coronary artery disease, stroke, Parkinson and chronic pulmonary disease. Factors affecting the nucleic acid shedding time, including vaccination status, the initial SARS-CoV-2 RT-PCR tests [N: 28.88 (2.78) vs. 28.62 (2.97), *P* = 0.656; ORF: 28.29 (2.97) vs. 27.86 (3.36), *P* = 0.501, respectively], severity on first admission, time from onset to enrollment in patients [1 (0–5) vs. 1 (0-5), *P* = 0.147], and medication were no significant different between the Paxlovid group and control group (Table 1).

**FIGURE 1:**
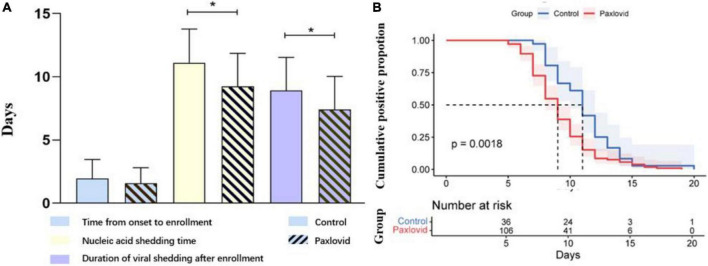
**(A)** Paxlovid can significantly reduce the nucleic acid shedding time of patients. No significant difference was find in the time from onset to enrollment between control and paxlovid group. **(B)** The nucleic acid shedding time of patients was calculated by Kaplan-Meier method. Mean ± SD. *P < 0.01.

In terms of the nucleic acid shedding time, the time to negative results was 11.11 (2.67) days in the control group and 9.32 (2.78) days in the Paxlovid group, respectively (Table 3 and Figure 1). Additionally, there was no significant difference in the time from onset to enrollment in patients between the two groups. Compared to the control group, results show that the shedding time was shorter in the Paxlovid group (*P* = 0.0018). For safety, no serious adverse events, severe cases, and death were reported after enrollment in either group. In the Paxlovid group, 28 people reported bitter mouth, accounting for 26.42%, but not in the control group.

**TABLE 3:** Outcomes in patients with infected with SARS-CoV-2 omicron.

Characteristic	Control group (*N* = 36)	Paxlovid group (*N* = 106)	*P*-value
Duration of viral shedding after enrollment, mean (SD), day	8.92 (2.61)	7.51 (2.79)	0.009
Nucleic acid shedding time*, mean (SD), day	11.11 (2.67)	9.32 (2.78)	0.001
Time from symptom appearance to disappearance, mean (SD), day	5.64 (2.87)	4.81 (3.00)	0.264
Death after enrollment, n (%)	0	0	–
Conversion to severe case after enrollment, n (%)	0	0	–
**Laboratory results^#^**			
WBC, mean (SD), (3.5–9.5 × 109/L)	6.32 (2.09)	6.43 (3.21)	0.867
L, mean (SD), (1.1–3.2 × 109/L)	1.49 (0.54)	1.61 (0.71)	0.413
Hemoglobin<130 g/L, n (%)	14 (48.28%)	35 (56.45%)	0.466
Platelet count <125 × 109/L, n (%)	0 (0.00%)	6 (9.09%)	0.093
Albumin <35 g/L, n (%)	21 (80.77%)	37 (63.79%)	0.120
CRP>10 mg/L, n (%)	9 (33.33%)	16 (25.40%)	0.441

WBC, White blood cell count; L, Lymphocyte count; CRP, C-reactive protein. *The nucleic acid shedding time was defined as first positive nucleic acid test to the date of the first negative test (in two consecutive). ^#^ Percentages may not total 100 in laboratory results because some patients did not receive blood sampling examination at discharge. The main reason was that the patient refused to take blood for examination after the symptoms improved significantly.

## Discussion

This non-randomized trial aims to assess the safety and efficacy of Paxlovid to treat in Chinese elder patients infected with SARS-CoV-2 omicron variants (age ≥ 60, mild or moderate cases). The nucleic acid shedding time was 11.11 days in the control group and 9.32 days in the Paxlovid group, respectively (*P* = 0.0018). In all participants, no cases of deaths and serious events were reported.

As of May 29, 2022, there are 568,716 asymptomatic carriers, and 57,980 cases were confirmed. Among the Chinese elder patients infected with SARS-CoV-2 omicron variants between February 26 to May 29, 2022, 588 (0.09%) people died, and 713 (0.114%) were severe cases. Compared with Wuhan in 2020, the severity rate and mortality rate of the epidemic in Shanghai are lower, while the infection rate is much higher (22). These results further confirmed that omicron variants had the characteristics of high infectivity and low virulence (14). In addition, the patients with severe cases were mainly older people, and the average age of death was 82.73. Based on this background, we conducted a non-randomized controlled trial aimed to explore the characteristics of Chinese elder patients infected with SARS-CoV-2 omicron variants and evaluated the therapeutic effect of Paxlovid.

Firstly, we analyzed 142 participants with omicron infection from the symptoms, serum indicators, and vaccination status. Like previous variants (23), fever, cough, and expectoration are also the first symptoms of the patient infection with omicron. 120 (84.51%) patients had a cough in this study, followed by 95 (66.90%) patients with expectoration. Our data show that patients with mild 118 (83.10%) were higher than patients with moderate 24 (16.90%), which once again emphasized that the majority of patients infected with omicron variant were asymptomatic and mild cases. The results of vaccination status revealed that the vaccination rate of patients over 60 years old was only 66.90%. Secondly, we analyzed the laboratory results. We found that 42 (29.58%) patients had a decrease in the WBC, and 51 (35.92%) patients had a decrease in the lymphocyte at the first admission. Moreover, there were 14 (9.86%) patients with albumin reduction at first admission.

At present, the prevention and treatment of COVID-19 is still a severe problem that needs to be solved urgently by people all over the world. Therefore, the specific drugs for COVID-19 still need to be further studied and updated. Paxlovid received the EUA for the treatment and has been proven effective against SARS-CoV-2 omicron variant infection (21). In a double-blind, randomized, controlled trial, the efficacy associated with the use of nirmatrelvir plus ritonavir among non-hospitalized, symptomatic adults with COVID-19 who were at high risk for progression to severe disease were evaluated. Their data show that treatment with nirmatrelvir early in COVID-19 can decrease progression to severe disease and reduce SARS-CoV-2 viral load (24). Currently, the SARS-CoV-2 omicron variant has become the predominant variant circulating in the world. However, the clinical studies on the efficacy of Paxlovid in patients infected with the SARS-CoV-2 omicron are still lacking. Therefore, to evaluate the efficacy of Paxlovid in treating Chinese elder patients infected with SARS-CoV-2 omicron variants, we enrolled 142 participants in the Paxlovid and control group according to the inclusion criteria and Chinese guidelines (version 9). No significant differences were found in the baseline characteristics. We further observed the therapeutic effect of Paxlovid in terms of nucleic acid shedding time and severe cases rate. Remarkably, Paxlovid can significantly shorten the nucleic acid shedding time of patients compared to the control group [9.32 (2.78) vs. 11.11 (2.67), *P* = 0.0018], respectively. None of the patients had severe or death cases. Additionally, no serious adverse events were recorded. Nevertheless, in the Paxlovid group, 28 people reported bitter mouth, accounting for 26.42%. This phenomenon should be paid attention to in future treatment.

Our trial also has limitations. (1) Patients who were treated with Paxlovid or not were based on the guideline rather than randomization. (2) Our trial is a single study with a small sample size. The number of the control group is lower than that of the Paxlovid group, which may reduce the power. (3) Participants were only COVID-19 patients aged 60 and over (mild or moderate cases). Therefore, the results presented in the data can only represent this part of the population and cannot wholly equal all patients infected with omicron. (4) The serological changes were partly affected by age and comorbidity. (5) We adopted nucleic acid shedding time to evaluate the effectiveness of Paxlovid, but not everyone was diagnosed on the first day. (6) Mahrokh et al. considered that prescribers might not be familiar with these drugs (19). This may lead to differences in treatment regimens. Given these complexities, they provided a step-by-step guide in managing patients with COVID-19 by Paxlovid as one of these effective drugs. In future studies on COVID-19, we can adopt this guideline to standardize the treatment methods of researchers.

## Conclusion

Our results demonstrate once again that omicron caused less severe cases of death but more infections. In our trial, patients’ first symptoms were mainly cough, fever, and expectoration. The laboratory results at the first admission showed that patients had the number of lymphocytes and leukocytes decreased. Our data also suggest that Paxlovid can significantly reduce the nucleic acid shedding time of patients. However, patients treated with Paxlovid may have a bitter mouth, which should be paid attention to in the later application. With a larger sample, future trials may further help to clarify the efficacy and safety of Paxlovid in Chinese patients infected with SARS-CoV-2 omicron variants.

## Data availability statement

The raw data supporting the conclusions of this article will be made available by the authors, without undue reservation.

## Ethics statement

The studies involving human participants were reviewed and approved by the Ethics Committee of the Ninth People’s Hospital, Shanghai Jiao Tong University School of Medicine (No. SH9H-2022-T112-2). Moreover, it was registered at the Chinese Clinical Trial Registry (ChiCTR2200060700). The patients/participants provided their written informed consent to participate in this study.

## Author contributions

WJZ, XJ, XY, ZD, WW, ZS, WCZ, LC, and YL collected the epidemiological and clinical data. WCZ and YL were responsible for enrollment, clinical monitoring, funding, study conception and design, and revising and submitting the final manuscript. XY, ZD, XJ, and YL were responsible for the distribution and storage of medicines. WJZ, TF, XN, LC, CZ, YL, and WCZ were responsible for statistical data. WJZ, XJ, XY, TF, CZ, and YL drafted the manuscript. All authors contributed to the article and approved the submitted version.
